# Pattern and Visual Prognostic Factors of Behcet’s Uveitis in Northwest Iran

**DOI:** 10.18502/jovr.v17i2.10796

**Published:** 2022-04-29

**Authors:** Leila Alizadeh Ghavidel, Masood Bagheri, Farideh Mousavi, Leila Rezaei, Somayyeh Hazeri, Hesam-Sadat Hashemi

**Affiliations:** ^1^Cornea & External Eye Diseases, Department of Ophthalmology, Royal Victoria hospital, McGill University, Montreal, Canada; ^2^Vitreoretina Surgery, Department of Ophthalmology, Imam Khomeini Eye Center, Kermanshah University of Medical Sciences, Kermanshah, Iran; ^3^Vitreoretina Surgery, Department of Ophthalmology, Nikookari Eye Center, Tabriz University of Medical Sciences, Tabriz, Iran; ^4^Biologist, Department of Biology, Concordia University, Montreal, Canada; ^5^Department of Ophthalmology, Nikookari Eye Center, Tabriz University of Medical Sciences, Tabriz, Iran

**Keywords:** Behcet's Disease, Behcet's Syndrome, Behcet's Uveitis, Iran, Prognosis, Uveitis

## Abstract

**Purpose:**

To investigate the pattern of ocular involvement in Behcet's disease (BD) with predictors of patients' final state of vision.

**Methods:**

This historical cohort encompassed the clinical records of 200 patients diagnosed according to the International Criteria for BD (ICBD), over a period of 17 years between 2004 and 2021.

**Results:**

The prevalence of Behcet's uveitis (BU) was more common in females and patients in the fourth decade of life. Ninety-five patients (47.5%) had evidence of ocular involvement in the initial ophthalmologic evaluation, and 171 patients (85.5%) manifested evidence of BU during the follow-up visits of which bilateral non-granulomatous panuveitis was the most common anatomical pattern of involvement (32.9%) followed by posterior (27.6%), anterior (26.5%), and intermediate (13.8%) uveitis. The prevalent accompanying signs were oral aphthous (67%), skin lesions (29%), and genital ulcers (19.5%). Cystoid macular edema (CME) was the most frequent ocular complication (62%), followed by cataract (57.5%) and epiretinal membranes (ERM) (36.5%). Univariate analysis showed the following determinants: male gender, younger age at onset, panuveitis, posterior uveitis, retinal vasculitis, and longer duration of uveitis as poorer visual prognostic factors of the disease. Multivariate analysis demonstrated a higher chance of poor visual prognosis of BD in patients with panuveitis, posterior uveitis, retinal vasculitis, and longer duration of uveitis.

**Conclusion:**

This cohort study demonstrated an overview on epidemiological patterns of BU along with the visual prognostic factors in Iranian patients.

##  INTRODUCTION 

Behçet's disease (BD) is a chronic, multisystem autoimmune vasculitis of unknown etiology with a wide range of clinical presentations, distinctive geographic distribution, and remarkable genetic background.^[1]^ Diagnosis is made clinically, characterized by relapsing oral–genital aphthous lesions, uveitis, and skin lesions. In addition, BD may involve multiple systems including pulmonary, musculoskeletal, gastrointestinal tract, and nervous systems.^[2]^

Behçet's disease is prevalent in the Mediterranean, Middle East, and Far East regions, an area encompassing countries along the ancient Silk Road (Iran, Turkey, China, Japan, Saudi Arabia, and Greece) with the highest human leukocyte antigen-B51 (HLA-B51) prevalence.^[3]^ It commonly involves the younger population often in the third and fourth decades of life with a male/female ratio of more than one.^[3]^ Although etiopathogenesis of BD has not yet been clarified, both immunogenetic susceptibility along with environmental predisposing factors have been implicated in its evaluation.^[2]^ HLA-B51 is the most well-known immunogenetic association link with BD and some studies have reported that ocular involvement is prevalent in HLA- B51-positive patients.^[4,5]^

Ocular involvement in the form of recurrent severe uveitis has poor prognosis, potentially culminating in irreversible visual loss despite recent dramatic advances in diagnostic–therapeutic measures. Behcet's uveitis (BU) is characterized by bilateral recurrent non-granulomatous panuveitis and occlusive retinal vasculitis, however, anterior, intermediate, or posterior uveitis can also occur.^[2]^ Posterior segment involvement has been reported in 50–93% of cases and relapsing uveitic attacks in the posterior segment may cause blindness due to irreversible retinal damage leading to atrophic maculopathy, optic atrophy, macular scarring,^[2]^ and even blindness.

Although the treatment of many cases of noninfectious uveitis is similar, regarding BD, its distinction from other causes has therapeutic and prognostic implications, for example, the need for aggressive treatment with immunomodulator therapy in the earlier stages.^[6]^ It is difficult to define the visual outcome of a BD case because of its ophthalmic clinical variability, and the unavailability of a validated and widely accepted tool for prognostication. As it is one of the most prevalent etiologies of noninfectious uveitis and one of the most common sight-threatening systemic diseases,[7, 8] determining pattern of involvement along with predictors of the patients' final state of vision is critical for epidemiological planning, appropriate treatment, patient follow-up, and estimation of visual prognosis. This study evaluated the epidemiological pattern of BU with the respective visual prognostic factors over a 17-year follow-up period at a referral center in northwestern Iran.

##  METHODS

In this historical cohort, the clinical records of patients with BD who were referred to the uveitis clinic of a tertiary ophthalmology hospital over the last 17 years were reviewed. According to the existing protocol defined in clinics at Tabriz University of Medical Sciences, all patients with a diagnosis of BD, based on the International Criteria for BD (ICBD),^[9]^ were referred to our ophthalmology center, Nikookari Eye Care Hospital, for evaluation and regular follow-up at the uveitis clinic. These patients formed the statistical population in this cohort study. We included all referred patients to this interdisciplinary uveitis clinic between 2004 and 2018 in addition to a follow-up period of less than three years or irregular follow-up periods, incomplete file information, or ages < 16 years were excluded from the study.

General clinical variables extracted included age at diagnosis of BD and BU, the first ophthalmic consultation, the first ocular involvement, gender, accompanying symptoms, and the type of topical, local, or systemic treatments (steroids, anti-metabolites, or cytotoxic agents). Evaluation of accompanying symptoms at the first ophthalmologic visit included searching for systemic involvement (oral aphthous, genital ulcer, skin lesions, pulmonary, musculoskeletal, gastrointestinal tract, and nervous systems manifestations) and positivity for HLA-B5 and B51. Ophthalmologic variables extracted consisted of best-corrected visual acuity (BCVA), intraocular pressure (IOP), type of uveitis, clinical course of disease (acute, recurrent, or chronic), number of uveitic attacks per year, paraclinical imaging results, and ocular complications (cataract, glaucoma, retinal detachment, macular edema, optic atrophy, atrophic maculopathy, band keratopathy, and ocular hypotony). In all patients with the diagnosis of uveitis, fluorescein angiography (FAG) was performed to evaluate the presence of retinal vasculitis. In this study, we defined moderate visual loss (MVL) and severe visual loss (SVL) as BCVA ≤ 20/40 and ≤ 20/200, respectively.

Anatomical location of involvement was classified based on the Standardization of Uveitis Nomenclature (SUN) Working Group,^[10]^ which is widely accepted today and is now the standard required for publication of uveitis studies in the peer-reviewed journals.

All factors affecting vision in terms of predictive and prognostic factors were extracted from patients' records including general clinical variables, ophthalmologic variables, and accompanying findings. Finally, the data were analyzed using SPSS software version 20 (IBM Corp., Armonk, NY, USA). The data are presented using descriptive statistical methods (i.e., mean, standard deviation, frequency, and percentage). Normality of data was tested using the Kolmogorov–Smirnov test. To compare the data, ANOVA and Chi-square tests were used for all ratio and nominal variables, respectively. Cox regression analysis was used for multivariate evaluation of risk factors. A *P*-value < 0.05 was considered as statistically significant.

The current study was approved by the ethical committee of the Tabriz University of Medical Sciences (code IR.TBZMED.REC.1397.239).

##  RESULTS

**Figure 1:**
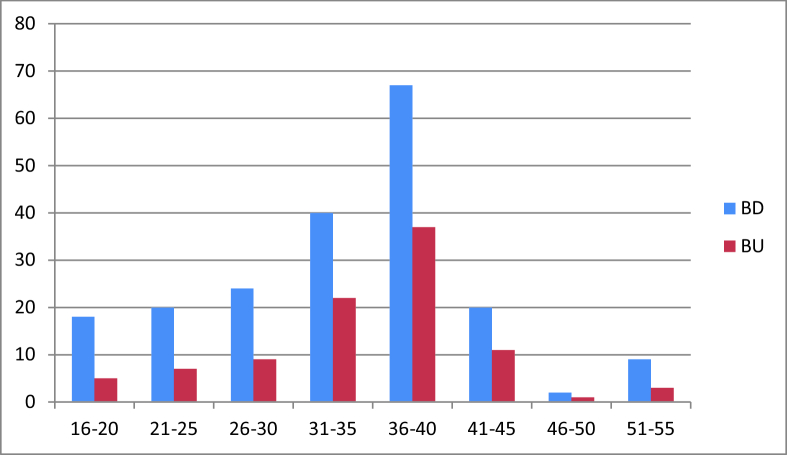
Frequency of BD (blue color) and BU (red color) in different age groups at first ophthalmologic visit. As shown, most patients with BU were in the age group of 31–40 years (62.1%). BD, Behcet's disease; BU, Behcet's uveitis.

In this study, we reviewed clinical records of 200 patients with BD who were referred to the uveitis clinic during the last 15 years from 2004 to 2018. The mean time interval between the diagnosis of BD and referral to the uveitis clinic was 28 days (range, 2–70 days). Of the 200 patients, 72 were male and 128 female with a male/female ratio of 0.56. The mean age of the patients at the time of diagnosis of BU was 33.09 ± 8.06 years for the cohort, 31.31 ± 7.21 years for males, and 36.01 ± 10.12 years for females; the youngest and oldest patients were 16 and 52, respectively. Ninety-five patients (47.5%) had evidence of ocular involvement at the initial ophthalmic evaluation. Most patients with BD and BU were in the age group of 31 to 40 years (53.5% and 62.1%, respectively). Thus, the prevalence of BU was more common in the fourth decade of life and in the female gender (29 were male and 66 were female) with a male/female ratio of 0.44. Figure 1 shows the age distribution of patients with BD and BU.

**Figure 2:**
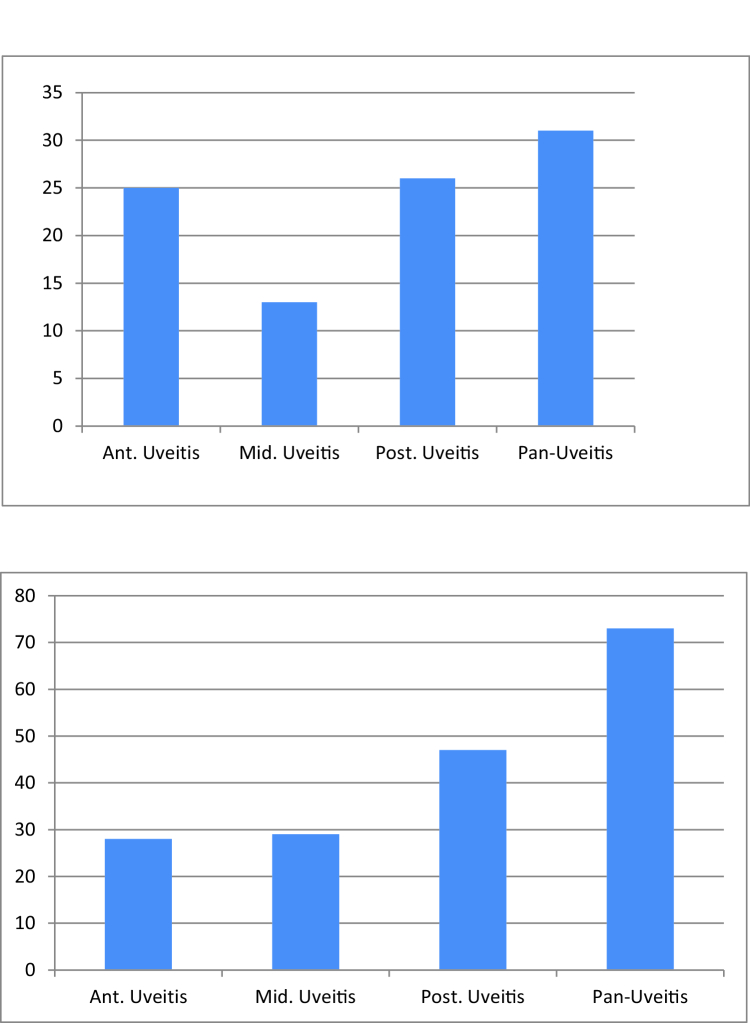
Frequency of uveitis type in study patients at first (left chart) and last (right chart) ophthalmic visits. As shown, panuveitis was the most common anatomical pattern of ocular involvement, followed by posterior uveitis. Ant. Uveitis, anterior uveitis; Int. Uveitis, intermediate uveitis; Post. Uveitis, posterior uveitis.

**Figure 3:**
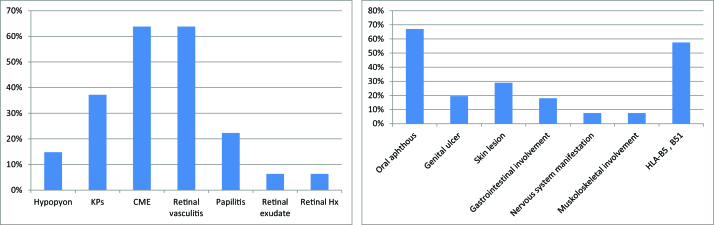
Frequency of ocular uveitic signs (left) and accompanying systemic symptoms and findings (right) at the first ophthalmologic visit in patients with BU. HLA, human leukocyte antigen.

**Figure 4:**
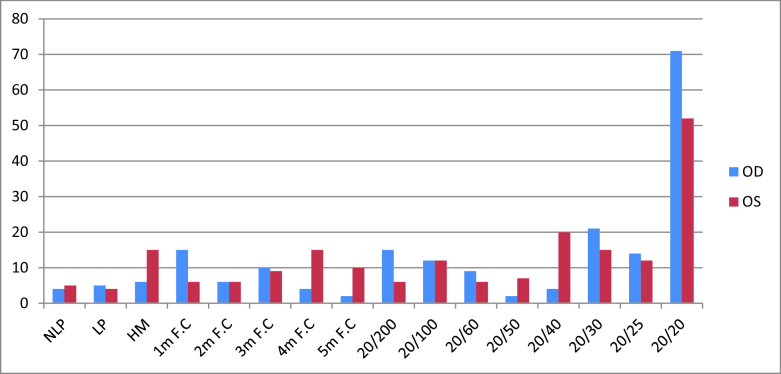
Frequency of the BCVA separately in the right (blue color) and left (red color) eyes at final ophthalmologic visit. The ophthalmologic examination of 400 eyes of 200 cases at the final visit showed that 9 eyes (2.25%) were no light perception (NLP), 9 (2.25%) had light perception (LP), and 123 (30.75%) had a 20/20 best corrected visual acuity (BCVA). Generally, frequencies of ≤ 20/40 and ≤ 20/200 BCVA at last follow-up were 53.75% and 35.75%, respectively. NLP, no light perception; LP, light perception; HM, hand motion; F.C, fingers counting.

**Figure 5:**
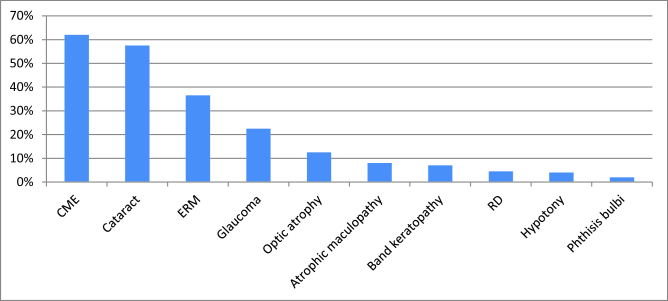
Frequency of ocular complications in patients with BU in the follow-up. As shown CME (62%) was the most common complication, followed by cataract (57.5%) and ERM (36.5%). (CME: cystoids macular edema, ERM: epiretinal membrane, RD: retinal detachment).

**Table 1:** Mean BCVA at the initial and last visits and the change in BCVA for different groups with the *P*-values of comparisons.


	**Initial BCVA**	**BCVA in last follow-up**	**Change in BCVA**	***P*** **-value**
Overall	0.39 ± 0.03	0.82 ± 0.05	0.43 ± 0.04	0.02
Male/Female	0.42 ± 0.03/0.33 ± 0.03	0.87 ± 0.06/0.72 ± 0.06	0.45 ± 0.05/0.39 ± 0.04	0.02
Ant. uveitis	0.45 ± 0.04	0.37 ± 0.03	-0.08 ± 0.03	0.61
Middle uveitis	0.43 ± 0.04	0.48 ± 0.04	0.05 ± 0.04	0.73
Post. uveitis	0.27 ± 0.02	1.03 ± 0.09	0.76 ± 0.07	0.00
Panuveitis	0.35 ± 0.03	0.97 ± 0.08	0.62 ± 0.05	0.01
Retinal vasculitis	0.40 ± 0.03	1.02 ± 0.07	0.62 ± 0.04	0.01
Macular inflammatory exudate	0.57 ± 0.03	0.97 ± 0.07	0.40 ± 0.05	0.02
Macular hemorrhage	0.60 ± 0.03	1.03 ± 0.10	0.43 ± 0.06	0.02
Hypopyon	1.05 ± 0.08	0.73 ± 0.05	-0.32 ± 0.06	0.02
KP	0.57 ± 0.05	0.43 ± 0.04	-0.14 ± 0.05	0.06
CME	0.87 ± 0.07	0.57 ± 0.04	-0.30 ± 0.05	0.03
Papillitis	0.92 ± 0.08	0.72 ± 0.06	-0.20 ± 0.07	0.04
	
	
BCVA, best corrected visual acuity; Ant. Uveitis, anterior uveitis; Mid. Uveitis, middle uveitis; Post. Uveitis, posterior uveitis; KP: keratic precipitate; CME, cystoids macular edema				

Of the 400 eyes of 200 cases, one eye (0.25%) had no light perception (NLP), one (0.25%) had light perception (LP) and 127 eyes (31.75%) had 20/20 BCVA at presentation. Frequencies of ≤ 20/40 and ≤ 20/200 BCVA at the first ophthalmic visit were 37.75% and 16.5%, respectively. The mean IOP was 13.7 ± 2.90 and 12.3 ± 2.30 in the right and left eyes, respectively (range, 10–25 mmHg). Out of the 95 patients with BU, 80 (85.1%) were bilateral and panuveitis was the most common type of anatomical pattern of involvement with a prevalence of 32.9%, followed by posterior (27.6%), anterior (26.5%), and intermediate (13.8%). Figure 2 shows the frequency of different types of uveitis at the initial ophthalmologic visit. In patients with ocular involvement, 60 (63.8%) had cystoid macular edema (CME), 60 (63%) had retinal vasculitis (macular, peripheral, or optic disc), and 14 (14.8%) had hypopyon. Retinal vasculitis was most commonly seen in panuveitis (29 cases, 39.72%), followed by posterior (18 cases, 38.29%), intermediate (9 cases, 31.03%), and anterior (4 cases, 14.28%) BU. The prevalence of uveitic ocular manifestations at the first ophthalmic visit is shown in Figure 3.

The prevalent accompanying symptoms were oral aphthous with a frequency of 67%, followed by skin lesions (29%) and genital ulcer (19.5%); HLA-B5 or B51 was positive in 115 cases (57.5%). Based on dermatology consults, the most common skin lesions included the following: papulopustular lesions (37 cases 18.5%); followed by reactivity of the skin to needle prick or injection (pathergy reaction) (35 cases, 17.5%); erythema nodosum-like lesions (31 cases, 15.5%); superficial thrombophlebitis (14 cases, 7%); furuncles (7 cases, 3.5%); extragenital ulceration (6 cases, 3%); pyoderma gangrenosum-like (4 cases, 2%); erythema multiforme-like lesion (3 cases, 1.5%); hemorrhagic bullae (2 cases, 1%); and abscesses (one case, 0.5%). Figure 3 illustrates the most common systemic presentations in this cohort of patients.

At the time of referral, only 2 (1%) cases were on no systemic therapy, 188 (94%) were on systemic corticosteroid therapy, and 173 (86.5%) were receiving immunomodulatory treatment, which included antimetabolites, cytotoxic agents, or biologic response modifiers in 131 (65.5%), 18 (9%), and 24 (12%) cases, respectively. Thirty-five (17.5%) patients were on monotherapy (25 with steroids and 10 with immunomodulatory), and the majority of patients (147, 73.5%) were on dual therapy with prednisone and immunomodulatory agents. Sixteen (8%) patients were receiving triple therapy with prednisone, azathioprine, and cyclosporine.

Eyes with macular involvement (including inflammatory exudates in 6 eyes, hemorrhage in 6 eyes, and CME in 60 eyes) or anterior uveitis accompanied with hypopyon (14 eyes) at presentation had the worst baseline BCVA compared to eyes without macular involvement (*P* = 0.01) or hypopyon (*P* < 0.001) which had the best BCVA at presentation.

Patients were followed for 7.3 years and the minimum and maximum follow-up periods were 3 and 17 years, respectively. During the follow-up period, all patients with BU underwent topical or local treatment to control active disease, and with the interdisciplinary cooperation, systemic medication doses were adjusted or changed by rheumatologists. During the follow-up ophthalmic visits, 171 patients (85.5%) subsequently manifested evidence of ocular involvement. Of the 400 eyes at the final visit, 9 (2.25%) had NLP, 9 (2.25%) had LP, and 123 (30.75%) had a 20/20 BCVA [Figure 4]. The frequencies of MVL and SVL at the last follow-up were 17.75% and 35.75%, respectively. In addition, 42 (29.16%) and 63 (43.75%) eyes of males and 29 (11.32%) and 80 (31.25%) eyes of females were affected by MVL and SVL, respectively, both being significantly higher in males (*P* = 0.01 and 0.02, respectively). The mean IOP of patients was 15.82 ± 3.60 and 15.3 ± 4.35 mmHg in the right and left eyes, respectively (range, 0–31 mmHg). Similar to the occurrence at the initial visit, bilateral non-granulomatous panuveitis was the most common pattern of manifestation during the follow-up, followed by posterior, anterior, and intermediate uveitis in order of prevalence [Figure 2]. All cases had bilateral and non-granulomatous manifestations but no unilateral or granulomatous involvement was seen. Males were more likely to experience pan or posterior uveitis in addition to both MVL and SVL over time.

In total, 142 patients (250 eyes) developed 630 complications during the follow-up period. CME was the most frequent (62%), followed by cataract (57.5%) and epiretinal membranes (ERM) (36.5%). Detailed information about ocular complications in BU during follow-up is illustrated in Figure 5.

The mean difference in BCVA between presentation and the follow-up visits was greatest for eyes with retinal vasculitis, macular inflammatory exudates, and hemorrhage. Eyes with retinal vasculitis, retinal exudates, or hemorrhage presented as the first uveitic signs, experienced worse BCVA in follow-up compared to those with hypopyon, keratic precipitate (KP), CME, and papillitis [Table 1]. In addition, eyes with no uveitis at the first ophthalmologic visit had the best visual outcome in general (data not shown). Visual prognosis in univariate analysis reflected worse in male, younger age at onset, presence of retinal vasculitis, panuveitis, posterior uveitis, and longer duration of uveitis; however, the male gender and younger age were not significantly associated with a higher risk of SVL in multivariate analysis. Regression analysis demonstrated a higher chance of poor visual prognosis in patients with panuveitis (OR: 3.468 (1.338–8.989), *P* = 0.01), posterior uveitis (OR: 5.008 (1.625–15.432), *P* = 0.005), retinal vasculitis (OR: 3.825 (1.414–10.343), *P* = 0.008), and longer duration of uveitis (OR: 1.002 (1.001–1.003), *P* = 0.002). If we exclude the eyes with glaucoma and cataract, posterior and panuveitis have a significant correlation with the evolution of structural complications (data not shown). Several variables including patients' age (*P* = 0.7), the presence of systemic manifestation of BD at baseline visit (*P *= 0.7), and HLA-B51 (*P* = 0.3) were not associated with either a significant higher rate of SVL or poor visual prognosis.

Among ocular uveitic complications [Figure 5], atrophic maculopathy, optic atrophy, and hypotony had statistically significant association with SVL (*P* = 0.005, *P*= 0.005, and *P* < 0.001, respectively). Subgroup survival analysis of the risk of SVL for the patients over five-years follow-up was significantly different between patients diagnosed before 2010 and those diagnosed after 2014 with a lower rate of SVL (*P* = 0.043) in the latter group, however, there is no difference in visual outcomes of patients treated with conventional immunomodulatory therapy (IMT) versus biologic agents in this cohort (*P* = 0.058).

##  DISCUSSION

BD is a visually threatening inflammation with serious implications for the patient as its ocular complications may be irreversibly detrimental, particularly when ischemic complications occur.^[11]^ We performed this study to map the epidemiological pattern of ocular involvement of BD in northwestern Iran and determine causes of vision loss and significant impact on eyesight over time. We further aimed to elucidate the influence of each of the ocular complications on the final visual outcome in 400 eyes of 200 confirmed patients who were referred for ophthalmic counseling and were regularly followed-up afterward. The prevalence of ocular involvement in BD was 85.5% in this series, which is significantly higher than the 70% previously reported in other studies.[12, 13]

In this historical cohort study, BU occurred predominantly in the fourth decade of life with a male/female ratio of about 0.44. The most common anatomical pattern of involvement was bilateral non-granulomatous panuveitis and the prevalent uveitic ocular manifestations at the first ophthalmic visit were CME and retinal vasculitis. Overall, 35.75% of the eyes had SVL at presentation which correlated with the presence of macular inflammatory exudates or hemorrhage, CME, and the presence of hypopyon. The most common causes of irreversible SVL were atrophic maculopathy, optic atrophy, and hypotony. Visual prognosis evaluated through univariate analysis was worse under the following variables: male, younger age at onset, presence of retinal vasculitis, panuveitis, posterior uveitis, and longer duration of uveitis; however, multivariate analysis demonstrated that male gender and younger age variables were not significantly associated with a higher risk of SVL. This was due to the higher prevalence of posterior or panuveitis in men than in women and the longer duration of uveitis in the latter.

In this study, the prevalence of BU was evaluated higher in females than males, although contrary to the results of the majority of comparative reports,^[14]^ it was in line with alternate reports carried out in the US and Western Europe.[11, 15, 16] In addition, an epidemiologic study conducted over a period of 44 years from 1968 to 2011 by Accorinti et al demonstrated that the incidence of ocular BD is increasing in females.^[17]^ Despite the corroborating evidence to suggest the increase of ocular BD in females, there is a consensus regarding the poorer visual prognosis in males as is demonstrated in our report.^[11]^ The multivariate analysis of the current cohort explained the poor prognosis in men and the higher prevalence of posterior or panuveitis in this subgroup.

Taylor et al demonstrated male gender, unilateral uveitis, left-sided BU, non-white race, and the non-usage of biologic agents as predisposing factors for SVL at short- and long-term follow-ups, but ocular ischemic manifestation, age at diagnosis of BU, and duration of BD were not associated with SVL.^[11]^

A monocentric Italian study by Sota and colleagues found that the duration of BU for > 15 years, panuveitis, and positive HLA-B51 were predictors of long-term structural complications.^[18]^ Cataract was determined as the prevalent complication, followed by ERM and CME.

Another study by Celiker et al reported patients' age as the only visual prognostic variable in BU while receiving interferon alpha-2a.^[19]^ In their study, the male gender was found to be a poor prognostic factor in univariate analyses, but it was not statistically significant when evaluated through multivariate analysis. In a cohort study by Amer et al, posterior and panuveitis were also deemed potentially sight-threatening presentations of BU which needed aggressive immunosuppressive therapy.^[14]^ Subgroup survival analysis of our cohort demonstrated reduced risk of SVL in patients diagnosed after 2014 as compared to patients diagnosed before 2010 over five-years of follow-up, which may indicate better control of disease with newly emerged immunomodulatory treatments in recent years. Many studies also reported that early and aggressive immunomodulatory treatment and the use of biologic agents are crucial for preventing recurrences and improving visual prognosis.[2, 11]

More recently, in a cohort study by Taylor and colleagues, the risk of severe visual loss after 10-year follow-up was estimated to be 13%.^[11]^ BCVA of patients in the current study measured at both initial and follow-up visits was often worse when compared to other studies. Possible causes may be the delay in referral or diagnosis, and ophthalmic consultation immediately after diagnosis (before appearance of the treatment effects). On the other hand, our study was historical, so it encompasses elements of selection bias, given that the center is a tertiary referral center, most probably referred patients already had more severe disease with higher probability of ophthalmic involvement. Nevertheless, this study provides important findings regarding the epidemiological pattern of BD and highlights some visual prognostic factors of the disease.

In conclusion, in this cohort, the prevalence of ocular involvement in BD was 47.5% and 85.5% in the first and follow-up ophthalmic exams, respectively. It was also discovered that bilateral non-granulomatous panuveitis was the prevalent pattern of BU. Retinal vasculitis, posterior and panuveitis were revealed to be risk factors for SVL, and complications such as atrophic maculopathy, optic atrophy, and hypotony correlated with SVL. Although BU was more prevalent in women, males were more likely to have pan or posterior uveitis with poorer visual prognosis over time. Finally, our patients reported worse BCVA at initial and follow-up visits as compared to alternate studies which may be due to delays in diagnosis or later referral of patients. So, the need for earlier diagnosis along with more aggressive treatment are an important point in improving the visual prognosis of patient.

##  Financial Support and Sponsorship

Nil.

##  Conflicts of Interest

There are no conflicts of interest.
